# Using staffing ratios for workforce planning: evidence on nine allied health professions

**DOI:** 10.1186/1478-4491-10-2

**Published:** 2012-02-01

**Authors:** Linda Cartmill, Tracy A Comans, Michele J Clark, Susan Ash, Lorraine Sheppard

**Affiliations:** 1 School of Public Health, Queensland University of Technology, Victoria Park Rd, Kelvin Grove, 4059, Brisbane, Australia; 2 School of Medicine, Griffith University, University Drive, Meadowbrook, 4131, Brisbane, Australia; 3 Division of Health Sciences, School of Health Sciences, The University of South Australia, Adelaide, Australia; 4 School of Public Health, Tropic Medicine & Rehabilitation Sciences, James Cook University, Townsville, 4811, Australia

## Abstract

**Background:**

Modern healthcare managers are faced with pressure to deliver effective, efficient services within the context of fixed budget constraints. Managers are required to make decisions regarding the skill mix of the workforce particularly when staffing new services. One measure used to identify numbers and mix of staff in healthcare settings is workforce ratio. The aim of this study was to identify workforce ratios in nine allied health professions and to identify whether these measures are useful for planning allied health workforce requirements.

**Methods:**

A systematic literature search using relevant MeSH headings of business, medical and allied health databases and relevant grey literature for the period 2000-2008 was undertaken.

**Results:**

Twelve articles were identified which described the use of workforce ratios in allied health services. Only one of these was a staffing ratio linked to clinical outcomes. The most comprehensive measures were identified in rehabilitation medicine.

**Conclusion:**

The evidence for use of staffing ratios for allied health practitioners is scarce and lags behind the fields of nursing and medicine.

## Background

Health care worldwide is a large and expensive industry for which there are high consumer expectations. As health care increasingly relies on expensive technologies and drugs, governments are under mounting pressure to find ways to contain costs. Managing staff costs has emerged as a major area of focus [1,2]. It has become apparent that there are service over-laps [3,4], and that staff allocation must be evidence-based [5,6] and focused on the needs of the population serviced rather than continuing with traditional areas and modes of service [7-9]. The Australian Productivity Commission recently identified under-utilisation of the professional competencies of staff as an area of concern for the Australian health workforce [10].

When introducing new services and reviewing current service delivery models, managers must make decisions on what constitutes appropriate levels of staffing. Different methods such as ratios; where staff are provided in a set ratio per measure such as bed numbers or population; and staffing according to patient acuity (for acute care services) are methods used in health care services for determining appropriate staff levels. While models of this type have been used successfully with nursing [11-13], and medical specialty professions [14], what constitutes appropriate levels of staffing for allied health professionals (AHPs) is less clear.

An Australian study [15,16] reviewed a number of workload capacity measures for use in estimating allied health staff requirements. Measures were categorised into ratio-based, procedure-based, care-based and diagnostic or casemix-based methodologies. The authors concluded that while the procedure-based method was the most widely used, overall the methodologies used in allied health workforce planning were poorly substantiated and rudimentary in nature.

### Aims

The aim of this review was to identify what workforce ratios have been used in nine allied health professions and to identify whether these measures would be useful for planning allied health workforce requirements.

## Method

A systematic literature search was performed using medical, business and allied health databases to identify workplace ratios for nine identified allied health professions: audiology, dietetics and nutrition, exercise physiology, occupational therapy, podiatry, physiotherapy, psychology, social work, and speech pathology. It was anticipated few items would achieve level 1-3 evidence using recognised criteria [17]. Similarly, methodological diversity was expected. In order to capture all possible relevant material, searches were not limited to any particular study design. Material retrieved included controlled trials, narrative reviews, audits, opinions and editorials.

Articles were included if they were published between and including the years 2000-2008, were related to workplace ratios, were transferable to the Australian context (defined as being from a country meeting United Nations criteria for a developed economy [18]), and identified numbers of professionals per specified number of beds or outpatients. Articles were excluded if they were outside a developed world setting, conducted in a setting other than health care, were not in the English language, or were a paper concerning professions outside the nine identified allied health professions.

Medical subject headings (MeSH) were identified and selected in consultation with a specialist librarian. The following search terms were used: *health manpower *or *health care reform *or *health resources *or *health services research *or *government *or *personnel management *or *workload *or *workforce *or *time management *or *quality of health care*. These were used in conjunction with the term allied health and the names of the nine target allied health professions: *allied health *or audiolog* or *dietician *or *dietitian *or *nutritionist *or exercise physiolog* or occupational therap* or podiatr* or physiotherap* or physical therap* or *psychologist *or *social worker *or speech patholog* or speech therap* or *speech and language*.

The databases examined included: Medline, Cinahl, ABI/Inform, Apais Health, Business Source, Embase, Meditext, OT Seeker, Psychinfo, and Pedro. In addition, electronic searches were conducted of Australian Health Review, Cochrane Library Economic Evaluation Database, website of Public Health Research Unit for the United Kingdom National Health Service (NHS), and the Service Delivery and Organisation Programme of the National Institute for Health Research-a research institute associated with the NHS website for the Joanne Briggs Foundation.

Requests were made to the professional bodies of the nine professional associations in Australia for any written documentation on workplace ratios and their web-sites were checked for published information. A reference group for the project included six of the nine professional groups and members were asked to search informal and grey literature (e.g. government reports and profession specific reports) relevant to their profession. Manual searching of reference lists of key articles and items recommended by informal professional contacts and peers produced three additional relevant references.

**Figure 1:**
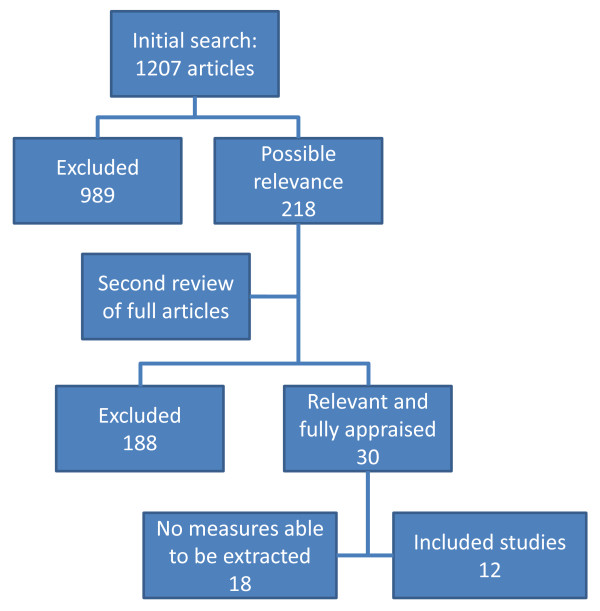
**Systematic literature search process**.

The initial strategy produced 1207 titles which were screened by title and abstract for compliance with inclusion and exclusion criteria; 989 papers were excluded. Where abstracts were not available or content uncertain, the full text article was obtained. 218 abstracts or full text articles were imported into Endnote. A second more detailed review was conducted and inclusion and exclusion criteria were applied independently by the two authors (LJC and TAC) from which 30 relevant articles were extracted. Exclusions were reviewed together and disagreements resolved by discussion after viewing the full text article where necessary. A version of the CriSTAL checklist for evaluating the quality of various research designs [19,20] was used to appraise included papers for their quality. After training in use of the appraisal tool, twenty articles were randomly selected and appraised by both LJC and TAC. An inter-rater correlation coefficient of 83% was achieved; LJC completed remaining appraisals. The review process is outlined in Figure 1.

Paper quality was determined by the presence or absence of study characteristics of: reliable data collection, adequate response rate, representativeness of participants, completeness of results, methodological limitations, and honest and objective conclusions.

## Results

The literature review identified 12 papers where figures were given for the ratio of different AHPs to bed or patient numbers. Table 1 briefly outlines the papers and gives the quality criteria score obtained from Section B of the CriSTAL checklist. Only one paper scored six out of a possible seven on study quality criteria [20]. Highly varied research methods and uncertain study quality were consistent across the professions. Physiotherapy was the most frequently recorded profession appearing in eight of the publications.

**Table 1:** Description of the included papers

Reference	Quality score*	Study Type	Country	Setting	Brief Description
AH Rehab CC (2007)[21]	1	Focus group	Australia	Rehabilitation	Consensus statement on required numbers of allied health to staff rehabilitation facilities treating various categories of patients

Aust. Fac. Rehab. Med. (2005) [22]	2	Non-systematic synthesis	Australia	Rehabilitation	Discussion paper estimating staffing numbers required for rehabilitation facilities

Br Diabetic Assn (1999) [29]	1	Position statement	Britain	Diabetes services	Provides recommendations for core staffing levels required for specialist diabetes services.

Burton et al (2005) [33]	2	Audit	Australia	Rehabilitation	Audit of psychologist numbers in ten hospitals in the rehabilitation sector of Victoria. All services reported under-staffing.

Christie (2006) [30]	2	Audit/benchmark	Canada	Acute hospital	Identification of physiotherapy and occupational therapy FTEs per bed over time in medical and surgical wards.

Gill (2007) [28]	2	Model	Australia	Chronic kidney disease	Model for provision of community based services for patients with chronic kidney disease via extended scope roles for dieticians and nurses.

Henley et al (2006) [25]	2	Non-systematic synthesis	Australia and New Zealand	Emergency department	Provides guidelines to the function and operations of acute MAPUs attached to hospital emergency departments.

McMillan & Ledder (2001) [23]	4	Audit	Britain	Neuro-rehabilitation	Survey of staffing in adult neuro-rehabilitation teams in south-east England. High incidence of staff stress due to the case-loads. Little is offered for those with psycho-social disability for example few teams contain neuro-psychologists.

Meyer et al (2002) [32]	6	Audit	Australia	All	University of Wollongong study of the dietetic workforce of New South Wales from 1984 to 2000.

Mudge, et al (2006) [24]	4	Controlled trial	Australia	Acute hospital	Controlled trial in an acute general setting of the effect of increased levels of multidisciplinary intervention. 1538 medical in-patients were assigned either to routine care or to care with three times the amount of allied health professional time. Resulted in significantly reduced in-hospital mortality and functional decline and improvement in patients' ratings of their health status

PA Hospital (2004) [26]	2	Pre-post study	Australia	Emergency department	In house survey of a trial of the use of AHPs (OTs, PTs and STs) in the Emergency Dept of a tertiary hospital for a period of 4 months over winter. Project made considerable cost savings for the hospital by avoiding in-patient admission for 117 patients over the period of the trial.

Ridoutt et al (2006) [15]	3	Literature review and focus group analysis	Australia	All	Aimed to identify current methods of quantifying AHP workload capacity and to profile the AHP workforce requirements. Recommended the use of a procedures based workload measurement tool. This tool would be useful in settings with set or routine treatments e.g. rehabilitation, community settings.

* CriSTAL Appraisal Score Section B (score out of 7)

AHP = allied health professional; FTE = full time equivalent; MAPU = medical assessment and planning unit; OT = occupational therapist; PT = physiotherapist; SP = speech pathologist.

Several papers demonstrated ratios by work setting rather than profession such as, adult rehabilitation [21-24] and emergency department [25,26]. Suggested ratios were also described for specific conditions, e.g. obesity [27] and kidney disease [28]. The ratios for each profession varied between the different settings. Additionally ratios varied between inpatient and community settings.

The methods applied in developing the ratios were derived from four main approaches: consensus, experimental trial, current clinical practice and those developed using staff classifications.

### Consensus

Consensus rather than observation or other means was used by six papers. Consensus came from working groups or committees. These papers presented standards [22], recommendations [21,25], and position statements [25,26,29] or benchmarks [30]. However, the ratios presented even varied within papers rather than clinical setting alone, with ratios varying to almost double [21,22], demonstrating the variability and difficulty of arriving at recommended ratios. Comparisons between recommended ratios within the same clinical setting, for example emergency departments [25,26], varied greatly. These papers highlight the difficulty of defining ratios without contextual role descriptions, for example, the roles of physiotherapists in the emergency department may vary from triage, undertaking first contact responsibilities or more conventional practice of referral from medical practitioners. In contrast, the Dieticians Association of Australia stated currently there is no standard to describe the ideal dietetic workforce in Australia [31].

### Experimental trial

Ratios derived from experimental trial were found in one paper. A controlled trial [24] studied the effects of a significantly increased level of interventions by AHPs. Outcome data of inpatient mortality, functional decline, patient reports of health status and length of stay were collected. The authors compared the increased cost of three times more AHPs with an unconvincing economic analysis of these outcomes to arrive at recommended ratios of AHP required for elderly patients with complex medical conditions.

### Current clinical practice

Current clinical ratios were collected by survey by dieticians [32] and psychologists [33]. Each reported current staffing rates as insufficient. An English paper surveying neuro-rehabilitation teams [23] also reported a high incidence of staff stress due to the size and nature of the case-load.

### Staff classifications

Finally, staff classifications were used to develop a ratio system which is independent of patient characteristics [15]. The staff classifications were used to present the possible clinical load as a percentage of full time equivalent (FTE) (85% reducing to 60%), with higher classifications or more experienced staff expected to have more managerial responsibilities and less clinical contact.

## Discussion

Table 2 indicates that there is a scarcity of research into staffing ratios for allied health professions. Only seven articles giving FTEs from functioning clinical examples could be located and potentially used in workforce planning. The usefulness of these is limited as they cover such a diverse range of settings and patient groups.

**Table 2:** Summary of the published ratios for allied health professionals

	Physio-therapist	Occ. therapist	Speech therapist	Social worker	**Clinical Psych**.	Neuro-Psych	**Dietitian**.	Podiat-rist
**Hospital Setting**								

Allied Health in Rehab. CC (2007) [21]								
Amputat.*	1.5	1.0	0.025	0.6	0.5	0.025	0.4	0.5
Arthritis	1.0	0.8	0.025	0.2	0.025	-	0.4	0.1
Burns*	2.0	2.0	0.2	1.2	1.0	-	0.4	0.025
Cardiac	0.75	0.5	0.025	0.25	0.025	-	0.4	0.1
Head Inj.*	1.5	1.5	1.5	1.2	0.2	1.0	0.5	0.025
Maj.Mult.Trauma	1.25	1.2	0.2	0.6	0.025	0.025	0.4	0.025
Neurol.	1.5	1.5	1.5	1.0	0.2	0.6	0.5	0.2
Orthopaed.	1.0	0.8	0.1	0.4	0.2	-	0.4	0.3
Pain	1.25	1.0	0.025	0.5	0.5	-	0.4	0.025
Pulmonary	0.75	0.75	0.1	0.25	0.025	-	0.4	0.1
Spinal*	2.0	2.0	0.25	1.2	0.5	0.1	0.4	0.2

Aust. Fac. R. Med. (2005) [22]								
Amputat. Acute	1.5	1.0	-		0.5	-		
Neurol. "	1.5	1.5	1.5		0.5	0.5		
Orthopaed. "	1.25	0.8	-		0.2	-		
Spinal "	2.0	2.0	0.25		0.5	-		
TBI "	1.5	1.8	1.5		0.7	0.5		
Amputat. Rehab	0.7	0.5	-		0.1	-		
Neurol. "	0.9	1.0	0.75		0.5	0.5		
Orthopaed. "	0.7	0.3	-		0.2	-		
Spinal "	0.9	1.0	-		0.5	-		
TBI "	0.9	1.5	1.0		0.5	0.5		

Burton (2007) [33]					0.15-0.3	0.05 to 0.5		

Christie (2006) [30]								
Gen. Surg.	0.3	0.1						
Orthopaed.	1.0	1.0						
Cardiovasc.	0.9	0.1						
Medical	0.5	0.3						
Rehab. (Geriatric)	0.5	0.3						
Rehab. (Stroke)	1.2	1.0						

Emergency Depts.								
Bris PAH (2004) [26]	2.5 AHP							
MAPU (Henley) (2006) [25] (25 bed)	0.25	0.5	0.2	0.5				

Meyer (2002) [32]								
Hosp. Urban/Rural							0.123/0.067	

Mudge (2006) [24]								
Complex Medical								
Intervention Gp.	0.4	0.4	0.2	0.4			0.16	
Usual Care Gp.	0.12	0.1	0.06	0.16			0.08	

Ridoutt (2006) [15] quoting Austin Hosp figures								
ICU	2.0							
Neurol.	0.9							
Gen. Rehab.	1.2 approx							
Unnamed Priv. Hosp All beds	0.92 AHP hrs/bd.dy							

**Community Setting**								

Br. Diabetes Assn. (1999) [29]/100 000							0.6	0.8

Gill (2007) [28]				1:70 pts	1:500 pts		1:80-120 pts 1:50-75 pts	

McMillan & Ledder (2001) [23]/100 000		1.5 community team professionals						

Meyer (2002) [32] Urban/Rural/100 000							0.093/0.082	

Figures are per 10 beds unless otherwise stated

* Denotes specialist unit

Within the Emergency Department area, for instance, it is difficult to compare the Princess Alexandra Hospital (PAH) figures [26] with those of the three described by Henley [25]. Where the PAH paper was of a four month trial over the winter period, the Henley paper describes the experiences of three diverse, ongoing, Medical Assessment and Planning Units (MAPUs). It is unclear how comparable in size are the Emergency Departments of PAH and the three hospitals of the Henley document; it is also unclear whether the clinical roles performed by the AHPs are comparable across sites for this new area of practice.

Of the five reports prepared by professional groups there are some similarities between the figures produced by the Allied Health in Rehabilitation group [21] and those produced by the Australasian Faculty of Rehabilitation Medicine [22]. It is noteworthy that the empirical Canadian experience [30] supports the Australian figures in the area of orthopaedics, while the Canadian figures for cardio-vascular wards are significantly lower than those recommended by the Allied Health in Rehabilitation group. However, without further information on the roles of staff or clinical outcome measures, it is not possible to adequately compare the ratios.

The Austin Hospital figures [15] for physiotherapy staff ratios enable comparison with those provided by the two professional groups in the area of neurology. The Austin figure of 0.9 FTE physiotherapists concurs with those of the Australian Faculty of Rehabilitation Medicine figures for rehabilitation neurology wards and is considerably less than the 1.5 FTE recommended by the Allied Health in Rehabilitation Collaborative Committee. It is uncertain if this represents different clinical practice or alternatively a difference of opinion between management and clinical therapists about appropriate staffing levels.

Only one paper [24] provided a link between staff FTEs and clinical outcomes, thus establishing an evidence base to support staffing numbers. The clinical intervention in that paper included increasing AHP staff, re-structuring teams and standardising communication systems. The results indicated a non-statistically significant trend towards a reduction in length of stay and in-patient bed-use which was seen as offsetting the cost of increased staffing levels. In addition, in-hospital mortality and functional decline were both significantly reduced.

While one paper [30] differentiated between AHP numbers required for general geriatric rehabilitation and stroke rehabilitation, there is no such division according to case-load in the figures provided by any of three other papers which also addressed rehabilitation staff ratios. Such inconsistencies make comparisons problematic.

## Conclusions

Use of staffing ratios to determine appropriate staff numbers can be a useful tool to guide service planning and delivery. This tool has been successfully used in nursing particularly in the acute care setting [13,14]. This review aimed to find out if allied health workforce ratios existed and if these ratios could be used in allied health service planning. However, there were few examples of staffing ratios found for AHPs and only one of these was a staffing ratio linked to clinical outcomes. From the papers found, it may be possible to apply ratios in the specific specialist areas of rehabilitation and MAPUs for allied health workforce planning. It is not possible from the evidence presented to use workforce ratios to plan for allied health requirements in general settings such as a general hospital or a community setting.

As the population ages and the incidence of chronic disease rises there will be increased demand for allied health services. Health managers and policy makers will need access to appropriate evidence based research to guide workforce planning to best meet community health needs. This review has found that research on staffing ratios for allied health practitioners is scarce and lags behind the fields of nursing and medicine. There is little data available on allied health requirements in general hospital settings such as orthopaedics and surgery and also in general community settings. This review highlights the need for further research on staffing ratios and their relationship to health outcomes across both hospital and community settings.

## Competing interests

The authors declare that they have no competing interests.

## Authors' contributions

LC conceived the research plan, undertook the systematic literature search and review and drafted the manuscript. TC conceived the research plan, undertook the systematic literature search and review and edited the final manuscript. MC, SA and LS contributed to the research plan and provided expert review and editing of the content. All authors approved the final manuscript.
